# Resveratrol Affects Cell Activities, Induces Apoptosis and Regulates AMPK Signaling Pathway in Pleural Mesothelioma Cells

**DOI:** 10.1096/fj.202500657RR

**Published:** 2025-10-19

**Authors:** Maria Rosa Iaquinta, Raffaella De Pace, Assia Benkhalqui, Cecilia Pesaresi, Simone Patergnani, Giulio Righes, Paolo Pinton, Mauro Tognon, Fernanda Martini, Elisa Mazzoni

**Affiliations:** ^1^ Department of Medical Sciences, Laboratories of Cell Biology and Molecular Genetics University of Ferrara Ferrara Italy; ^2^ Department of Translational Medicine University of Ferrara Ferrara Italy; ^3^ Department of Surgical Sciences, Dentistry, Gynaecology and Paediatrics University of Verona Verona Italy; ^4^ Department of Medical Sciences, Laboratory for Technologies of Advanced Therapies University of Ferrara Ferrara Italy; ^5^ Laboratory for Technologies of Advanced Therapies (LTTA) University of Ferrara Ferrara Italy

**Keywords:** AMPK, apoptosis, cell growth inhibition, invasion, pleural mesothelioma (PM), resveratrol (RSV)

## Abstract

Pleural Mesothelioma (PM) is an aggressive tumor with a poor prognosis and limited therapeutic options. Despite the notion that resveratrol (RSV) significantly inhibits the growth of PM cancer cells, it is necessary to evaluate the effects of this stilbene as a tumor suppressor agent. In this work, the effects of the natural polyphenol were investigated on PM cell lines (MSTO‐211H and IST‐MES 2) to evaluate its action as a potential adjuvant agent, together with chemotherapy. Our results showed that RSV treatment was effective in PM cell lines, in particular in MSTO‐211H. RES treatment decreases the viability evaluated with the MTT assay and Live/Dead staining. RSV stimulates the apoptotic process with positive staining for Annexin V‐PI and Caspase‐3/7 and inhibits the migration ability of both PM cell lines. In IST‐MES 2, RSV causes a reduction in mitochondrial and cytoplasmic calcium levels. Moreover, RSV affects cellular morphology, E‐cadherin protein expression, and decreased nuclear localization of β‐catenin, attenuating Wnt/β‐catenin signaling, which regulates tumor cell proliferation. At the molecular level, RSV modulated the expression of key genes that play an important role in cellular adhesion, proliferation, and metabolic activity, as well as AMPK signaling. RSV seems to be a promising therapeutic adjuvant agent for PM treatment.

## Graphical abstract

This graphical abstract presents the study of Resveratrol as a potential adjuvant therapy for Pleural Mesothelioma (PM), focusing on the epithelioid and biphasic histotypes using IST‐MES 2 and MSTO‐211H cell lines. Following 24 h of cell seeding, the cells were treated with Resveratrol, and various analyses were conducted to assess its effects. These included the evaluation of cell viability using MTT and Live/Dead assays, apoptosis and necrosis through Annexin V/PI staining and Caspase 3/7 activation, and cellular metabolism, focusing on calcium signaling, AMPK signaling, and real‐time PCR. Additionally, cellular adhesion was examined using wound healing assays, analysis of cell adhesion and ECM molecules via real‐time PCR, and immunofluorescence for proteins related to epithelial‐mesenchymal transition (EMT). The results suggest that Resveratrol holds significant potential as a novel therapeutic approach for PM, particularly for the biphasic histotype. The results highlight Resveratrol's promising role in improving PM treatment outcomes, offering a potential alternative to standard therapies like chemotherapy, surgery, and radiotherapy, which often yield unsatisfactory results.

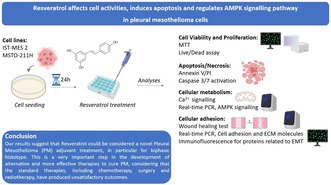

## 1 Introduction

Pleural Mesothelioma (PM) is a lethal cancer with an increasing incidence worldwide [1]. PM is the most common form of mesothelioma, accounting for over 90 000 deaths per year globally, with approximately 80% of cases caused by exposure to asbestos fibers [2]. The latency period for PM onset is approximately 20–40 years from the time of asbestos exposure to diagnosis, conferring a median survival of 8–14 months from diagnosis and a 5‐year survival rate of 5%–10% [3, 4]. The World Health Organization (WHO) Classification of Thoracic Tumors (fifth edition, 2021) [5] indicates that diffuse PM includes three major histotypes: epithelioid (70%–80% of cases), sarcomatoid (10%), and biphasic or mixed (10%–20%) [6]. The reported median overall survival rates for epithelioid, biphasic, and sarcomatoid PM are 19, 12, and 4 months, respectively [6, 7]. PM, which occurs after a long latency, is difficult to diagnose due to (i) cancer heterogeneity, (ii) distinct and non‐specific signs and symptoms, and (iii) diagnosis often assessed at an advanced disease stage, when in the worst case PM is already metastatic and considered unresectable. The prognosis of PM is very poor, and treatment options are far from satisfactory, with no single‐modality therapy proven effective due to chemo‐ and radiotherapy resistance. Asbestos exposure leads to the development of PM through multiple mechanisms, including chronic inflammation, oxidative stress, and persistent aberrant signaling, driving the transformation of normal mesothelial cells. Numerous studies have provided evidence suggesting that asbestos induces oxidative stress and inflammation, affecting acute respiratory diseases, lung cancer, and overall mortality [8]. The specific mechanisms through which inflammation affects the development of PM are not fully understood. Growing evidence supports a link between local and systemic inflammatory responses and patient prognosis [9]. Approved systemic treatments for PM have been limited to chemotherapy regimens that provide moderate survival benefits with poor outcomes [10]. Immune checkpoint inhibitors (ICIs) have shown promise over standard chemotherapy after more than a decade without novel treatment options, demonstrating better overall survival in both first‐ and later‐line settings. Still, a sizable fraction of patients does not respond to ICIs, underscoring the need for novel treatment approaches. The development of more potent treatments is necessary since standard medications have significant toxicity and their use is frequently linked to tumor resistance. It is important to explore the possibility of combining better‐tolerated compounds with conventional drugs and to devise synergistic treatments with reduced side effects and greater efficacy.

Recent years have seen the development of several alternative strategies based on natural substances, which have made significant advancements in cancer treatment possible. Plant polyphenols, including polyphenol‐rich extracts, display various biological activities including antioxidative, antiproliferative, pro‐apoptotic, anti‐angiogenic, and anti‐inflammatory properties [11, 12, 13]. Resveratrol (RSV) is a stilbene polyphenol found in several plant species and known for various potential beneficial effects. RSV has many biological and pharmaceutical properties, including antioxidant and antitumorigenic capabilities, including against PM [14, 15, 16, 17]. In this study, PM cell lines (MSTO‐211H and IST‐MES 2) were employed to evaluate in vitro the biological properties of RSV in terms of antiproliferative, pro‐apoptotic, and metabolic effects.

## 2 Materials and Methods

### 2.1 Cell Culture and Chemicals

Human mesothelioma cell lines MSTO‐211H (biphasic histotype) and human mesothelial cells (HMC) were obtained from the American Type Culture Collection (ATCC, Euroclone, Milan, Italy, catalog no. CRL‐2081). MSTO‐211H is a fibroblast‐like cell line isolated from the lung of a patient with biphasic mesothelioma. MSTO‐211H and HMC cells were maintained at 37°C in a humidified atmosphere with 5% CO_2_ in RPMI‐1640 (Euroclone, Milan, Italy) supplemented with 10% FBS (catalog no. ECS0180L, Euroclone, Milan, Italy) and 1% penicillin and streptomycin (P/S) (Lonza, Milan, Italy). IST‐MES 2 (epithelioid histotype), obtained from the GMP Cells and Cultures Bank, National Cancer Institute (ICLC, Genoa, Italy), were grown in DMEM F12 (Lonza, Milan, Italy, cat. no. BE12‐719F) supplemented with 10% FBS and 1% P/S. Cells were grown to 80% confluence in monolayer culture for 24, 48, and 72 h before treatments. RSV (Merck, Milan, Italy, cat. no. Y0001194) stock solutions were dissolved in double‐distilled water at a concentration of 100 μM.

### 2.2 Cell Viability Assay

MTT (3‐(4,5‐dimethylthiazol‐2‐yl)‐2,5‐diphenyl tetrazolium bromide) assay (Sigma‐Aldrich, Merck Life Science, Milan, Italy, cat. no. M5655) was performed to assess cell viability of PM cell cultures and HMC cells. Cells (5 × 10^3^ cells/well) were seeded onto 96‐well microtiter plates and treated with vehicle (medium) or various concentrations (1–1000 μM) of RSV for 24, 48, and 72 h. At the end of treatment, MTT solution was added to each well at a final concentration of 0.5 μg/mL. Viable cells contain NAD(P)H‐dependent oxidoreductase enzymes that reduce MTT to formazan. After 4 h incubation at 37°C, formazan crystals were solubilized using HCl‐acidified isopropanol. Absorbance was measured at 570 nm with a reference wavelength of 690 nm at room temperature using a spectrophotometer (Thermo Electron Corporation, model Multiskan EX, Finland). Relative cell viability (%) was expressed as a percentage relative to untreated cells. All experiments were performed in triplicate, and data are expressed as mean ± SD [18].

### 2.3 Live & Dead Staining Assay

Live/Dead Cell Double Staining Kit for mammalian cells (Merck, Milan, Italy, cat. no. QIA76) was used to analyze cell viability [18]. For each PM cell line and control, 5 × 10^4^ cells/well were plated in 24‐well culture plates and treated with 100 μM RSV for 24, 48, and 72 h at 37°C in a humidified atmosphere with 5% CO_2_. Live/Dead assay was performed according to the manufacturer's instructions. Cell‐permeable green fluorescent Cyto‐dye (Ex. max.: 488 nm; Em. max.: 518 nm) stained live cells, while propidium iodide (Ex. max.: 488 nm; Em. max.: 615 nm) stained dead cells. Digital images were acquired using a TE 200‐E fluorescent microscope through ACT‐1 software for DXM120F digital cameras (Nikon Instruments, Sesto Fiorentino, Italy) at 20X magnification and analyzed using ImageJ software. Analysis was performed in triplicate for each experimental group.

### 2.4 Annexin V/PI Staining

To evaluate the pro‐apoptotic effect induced by RSV in treated PM cells, Annexin V‐Propidium Iodide (PI) staining assays were performed using the Tali Apoptosis Kit—Annexin V Alexa Fluor 488 and Propidium Iodide (Life Technologies, Monza, Italy, cat. no. A10788) [18]. Cells (5 × 10^4^ cells/well) were seeded in 24‐well plates and treated with 100 μM RSV for 48 h. Then, cells were trypsinized and washed with 1× PBS, centrifuged at 8000 RPM for 10 min. After discarding the supernatant, cells were incubated with 100 μL Annexin Binding Buffer 1X (ABB) and 5 μL Annexin V Alexa Fluor 488 per sample for 20 min. Cells were then incubated for 3 min at room temperature with 1 μL of PI. Finally, cells were loaded into Tali Cellular Analysis Slides and analyzed using the Tali Image‐Based Cytometer. Apoptotic cells showed green fluorescence (early apoptosis), green and red fluorescence (late apoptosis), and necrotic cells showed red fluorescence.

### 2.5 Caspase‐3/7 Activated Protein Staining

Cell apoptosis was evaluated using Cell Event Caspase‐3/7 Green Detection Reagent (Life Technologies, Milan, Italy, cat. no. C10723) according to the manufacturer's instructions. This assay uses a fluorogenic substrate for activated caspases‐3/7. Upon caspase activation in apoptotic cells, the substrate is cleaved, enabling the dye to bind DNA and produce fluorescence. Cells (5 × 10^4^ cells/well) were seeded onto 24‐well plates and, upon reaching 80%–90% confluence, treated with 100 μM RSV. After 48 h, cells were stained with the green detection reagent diluted in 1× PBS and 5% FBS and incubated at 37°C for 30 min. Fluorescence intensity was recorded using DXM120F digital cameras (Nikon Instruments, Sesto Fiorentino, Italy) with excitation/emission at 530 nm.

### 2.6 Cellular Calcium (Ca^2+^) Homeostasis Measurement

This assay measures Ca^2+^ signaling in various cell types and subcellular compartments. The jellyfish *Aequorea victoria* produces a 22‐kDa protein named aequorin, which plays an important role in studying Ca^2+^ signaling [19]. Aequorin reacts with Ca^2+^ via oxidation of its prosthetic group, coelenterazine, resulting in light emission. Two chimeric aequorin probes were used: wild‐type cytosolic aequorin (cytAEQ) and mitochondrial‐targeted aequorin (mtAEQ, low‐affinity D119A mutant). The protocol included: PM cell line transfection, aequorin reconstitution, light recording during Ca^2+^ homeostasis perturbation, and light recording during cell lysis (to measure total aequorin amount).

Cells were seeded in 24‐well plates (2.5 × 10^4^ cells) on 12 mm slides and incubated at 37°C with 5% CO_2_ until 70%–90% confluence. Transfection was performed using Lipofectamine 2000 (Life Technologies, Milan, Italy) following the manufacturer's instructions [20]. Two mixes were prepared: (i) mitochondrial plasmid DNA (1.12 μg/μL) in serum‐free Opti‐MEM medium, and (ii) cytoplasmic plasmid DNA (1.8 μg/μL) in serum‐free Opti‐MEM plus Lipofectamine 2000. After 24 h, cells were treated with 100 μM RSV.

Aequorin measurements were taken 24 h after treatment. Cells transfected with cytAEQ and mtAEQ were reconstituted at 37°C and 5% CO_2_ for 1–2 h in Krebs‐Ringer buffer (KRB) supplemented with 1 mM CaCl_2_ and glucose (0.5 g in 500 mL). A 200 μL aliquot of complete KRB with 5 μM coelenterazine (Sigma‐Aldrich, Milan, Italy) was added to each well. Slides were placed in a temperature‐controlled perfusion chamber (37°C). Cells were perfused with KRB via a peristaltic pump. Luminescence was recorded using a low‐noise photomultiplier tube in a dark box until a stable signal was obtained. Then, cells were stimulated with 100 mM histamine to induce Ca^2+^ release from the endoplasmic reticulum. Finally, cells were treated with Triton X‐100 (Sigma‐Aldrich) to drain remaining aequorin. Data were collected and calibrated offline into [Ca^2+^] values using a computer algorithm based on aequorin Ca^2+^ response curves.

### 2.7 Wound‐Healing Assay

PM cell lines were seeded in 24‐well plates at 5 × 10^4^ cells/well and cultured at 37°C with 5% CO_2_ until reaching 90% confluence. A scratch was made using a 1000 μL pipette tip, then treated with 100 μM RSV. Images were acquired at 0, 24, 48, and 72 h using a bright‐field microscope (TE2000E Nikon s.p.a., Florence, Italy) at 4X magnification. Digital images were collected with ACT‐1 and ACT‐2 software for DXM1200F digital cameras (Nikon Instruments) [18]. Experiments were performed in triplicate. Cell migration was measured by comparing the scratch area before and after treatment using ImageJ software. Results were expressed as the average percentage of wound closure (%) up to 72 h.

### 2.8 Immunofluorescence

PM cells were seeded (2.5 × 10^4^ cells) on 12 mm slides in 24‐well plates and incubated at 37°C with 5% CO_2_. Upon reaching 70% confluence, cells were treated with 100 μM RSV. Control cells were grown in basal medium. After 48 h, slides were fixed in 4% paraformaldehyde for 20 min (Sigma‐Aldrich, Milan, Italy), followed by permeabilization with 0.1% Triton X‐100 for 10 min at room temperature. Immunofluorescence was performed to evaluate β‐Catenin, TGF‐β1, E‐Cadherin, and Vimentin expression.

Cells were incubated with mouse anti‐β‐catenin primary antibody (Thermo Fisher, Milan, Italy) at a 1:150 dilution in 1× PBS with 3% BSA for 1 h at room temperature (RT). After three washes with 1× PBS, cells were incubated with Alexa Fluor 488 anti‐mouse IgG secondary antibody (Thermo Fisher) at a 1:400 dilution in PBS with 3% BSA for 1 h at 37°C.

For other proteins, cells were incubated for 1 h at RT with rabbit anti‐TGF‐β, anti‐E‐cadherin, and anti‐Vimentin monoclonal primary antibodies (Thermo Fisher) at dilutions of 1:500, 1:200, and 1:300 in 1× PBS with 3% BSA, respectively [18]. After washing, cells were incubated with Alexa Fluor 488 anti‐rabbit IgG secondary antibody (Thermo Fisher) at a 1:200 dilution in 1× PBS with 3% BSA for 1 h at 37°C. Cell nuclei were stained with 0.5 mg/mL DAPI (Sigma‐Aldrich, Milan). Images were acquired using a fluorescence microscope (TE2000E Nikon s.p.a., Florence, Italy) at 20X magnification with ACT‐1 and ACT‐2 software for DXM1200F digital cameras (Nikon Instruments, Florence, Italy). Experiments were performed in triplicate.

### 2.9 qRT‐PCR for Human Extracellular Matrix, Adhesion Molecules and AMPK Signaling Pathways

The Human Extracellular Matrix (ECM) and Adhesion Molecules PCR Array (Qiagen, Milan, Italy, cat. no. 330231‐PAHS‐013ZA) and the AMPK Signaling Pathway PCR Array (Qiagen, Milan, Italy, cat. no. ID‐PAHS‐175Z) were employed to analyze gene expression modulation in PM cells treated with 100 μM RSV for up to 24 h. Total RNA was isolated using the RNeasy Mini Kit (Qiagen, Milan, Italy, cat. no. 74104) according to the manufacturer's protocol. RNA quality and quantity were assessed using a Nanodrop spectrophotometer (ND‐1000, Nanodrop Technologies, Wilmington, DE, USA). Subsequently, RNA was reverse‐transcribed into cDNA using the RT^2^ First Strand cDNA Kit (Qiagen, Milan, Italy) as recommended [21, 22]. The RT^2^ Profiler PCR Array for ECM and Adhesion Molecules enabled the analysis of 84 genes involved in cell‐to‐cell adhesion, cell‐to‐ECM adhesion, and included 5 housekeeping genes. The RT^2^ Profiler PCR Array for AMPK Signaling allowed the analysis of 84 genes related to IGF‐1 signaling, GSK3 inactivation, β‐Catenin accumulation, PTEN and apoptosis signaling, BAD phosphorylation and anti‐apoptotic pathways, mTOR signaling, as well as 5 housekeeping genes. Real‐time PCR analyses were performed using the CFX96 Touch PCR Detection System (Bio‐Rad, Milan, Italy) [21]. All analyses were conducted in triplicate for each experimental group.

### 2.10 Statistical Analysis

Statistical analysis was performed by one‐way analysis of variance (ANOVA) followed by *t*‐test with Bonferroni's post hoc test, based on *n* = 3 independent experiments each conducted in triplicate. Data analysis was performed using GraphPad Prism version 9.0 for Windows (GraphPad, La Jolla, CA, USA). *p* < 0.05 were considered statistically significant.

## 3 Results

### 3.1 RSV Activities on Cellular Proliferation and Viability

**FIGURE 1:**
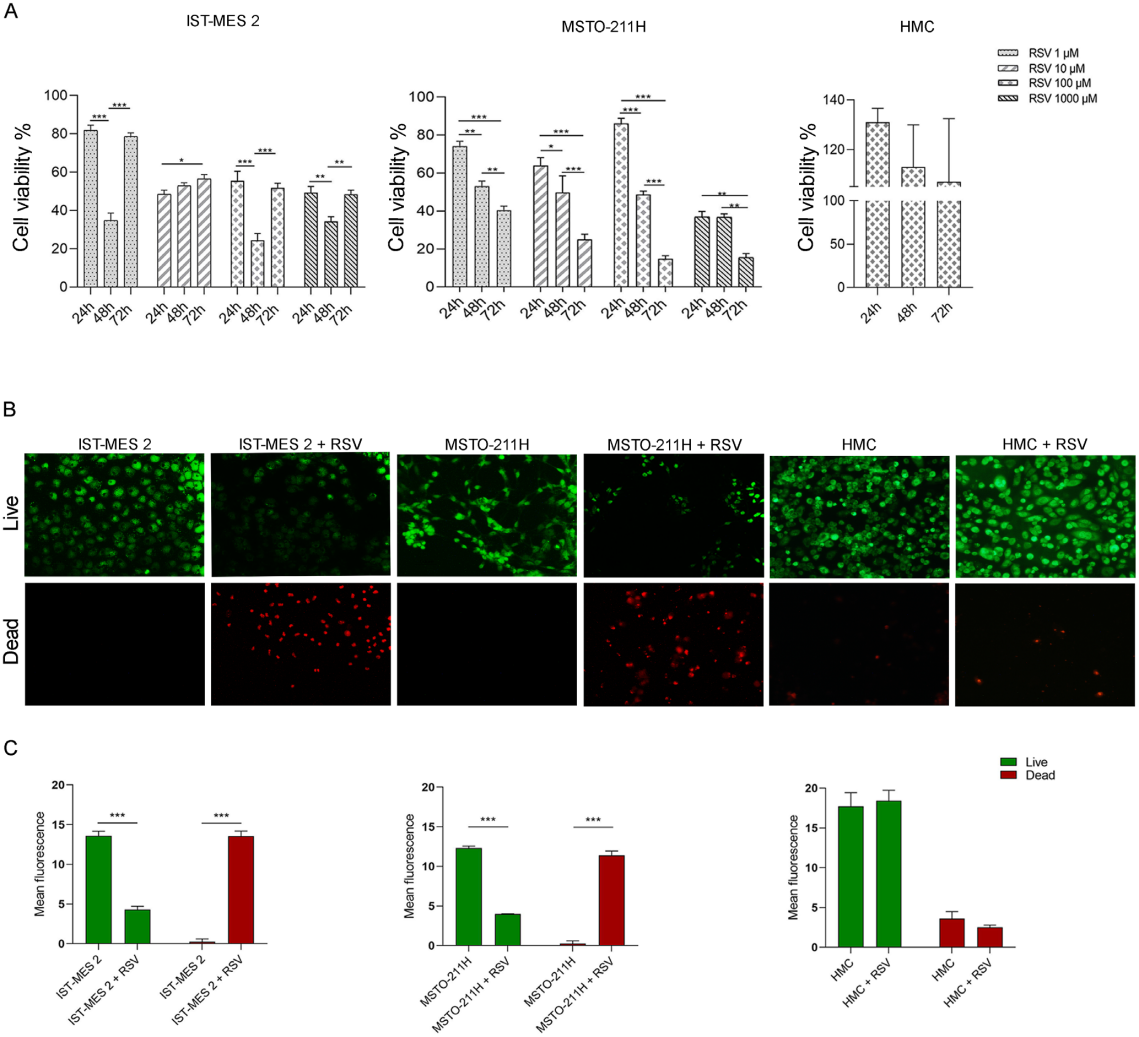
Effects of RSV on MSTO‐211H and IST‐MES 2 cell viability. (A) MTT evaluation of RSV (1–1000 μM) on MSTO‐211, IST‐MES and HMC cell lines at 24, 48, and 72 h. (*p < 0.05; **p < 0.001; ***p < 0.0001). (B) Live and Dead staining after RSV 100 μM (48 h) on MSTO‐211H, IST‐MES 2 cell lines, and HMC (control cells). Images acquired with fluorescence microscope, magnification 20X. (C) Quantification of mean fluorescence intensity with ImageJ software (*p < 0.0001).

MTT assay was performed to assess the effect of RSV on PM cell lines. MSTO and IST‐MES 2 cells were treated with different concentrations of RSV (1–1000 μM) for 24, 48, and 72 h. RSV at 100 μM inhibited MSTO‐211H proliferation as follows: RSV 1 μM (70%–44%), RSV 10 μM (60%–30%), RSV 100 μM (80%–15%), and RSV 1000 μM (50%–30%) during the 24–72 h incubation (Figure 1A). In IST‐MES 2 cells, a significant reduction in cell proliferation was observed with RSV at 1 μM (80%–50%), RSV 10 μM (50%–60%), RSV 100 μM (50%–30%), and RSV 1000 μM (50%–30%) during the 24–72 h incubation (Figure 1A). The results clearly showed that RSV was able to impair cell proliferation in both PM cell lines. Based on these data, the concentration of 100 μM was selected for subsequent analyses. RSV 100 μM was also used to treat HMC, demonstrating that this concentration did not affect HMC cell proliferation compared to the control (*p > 0.05, Figure 1A). Exposure to RSV significantly reduced MSTO‐211H and IST‐MES 2 cell viability in a dose‐dependent manner compared to the control group. RSV significantly inhibited PM proliferation at all time points investigated (Figure 1A).

Live and Dead analysis was used to confirm the cytotoxic effects of RSV (100 μM) on PM cells at 48 h. Cyto‐dye, a green fluorescent dye, and propidium iodide, a red fluorescent dye, were used to stain live and dead cells, respectively. Our data demonstrated that RSV was able to reduce PM cell viability, inducing cell death after 48 h of incubation. These data were further confirmed by fluorescence quantification shown in Figure 1C. Moreover, digital images showed the presence of live HMC cells after RSV treatment, which were indistinguishable from those grown under control conditions. Conversely, dead cells were not detected (Figure 1B,C).

### 3.2 RSV Induces the Apoptotic Process in PM Cell Lines

**FIGURE 2:**
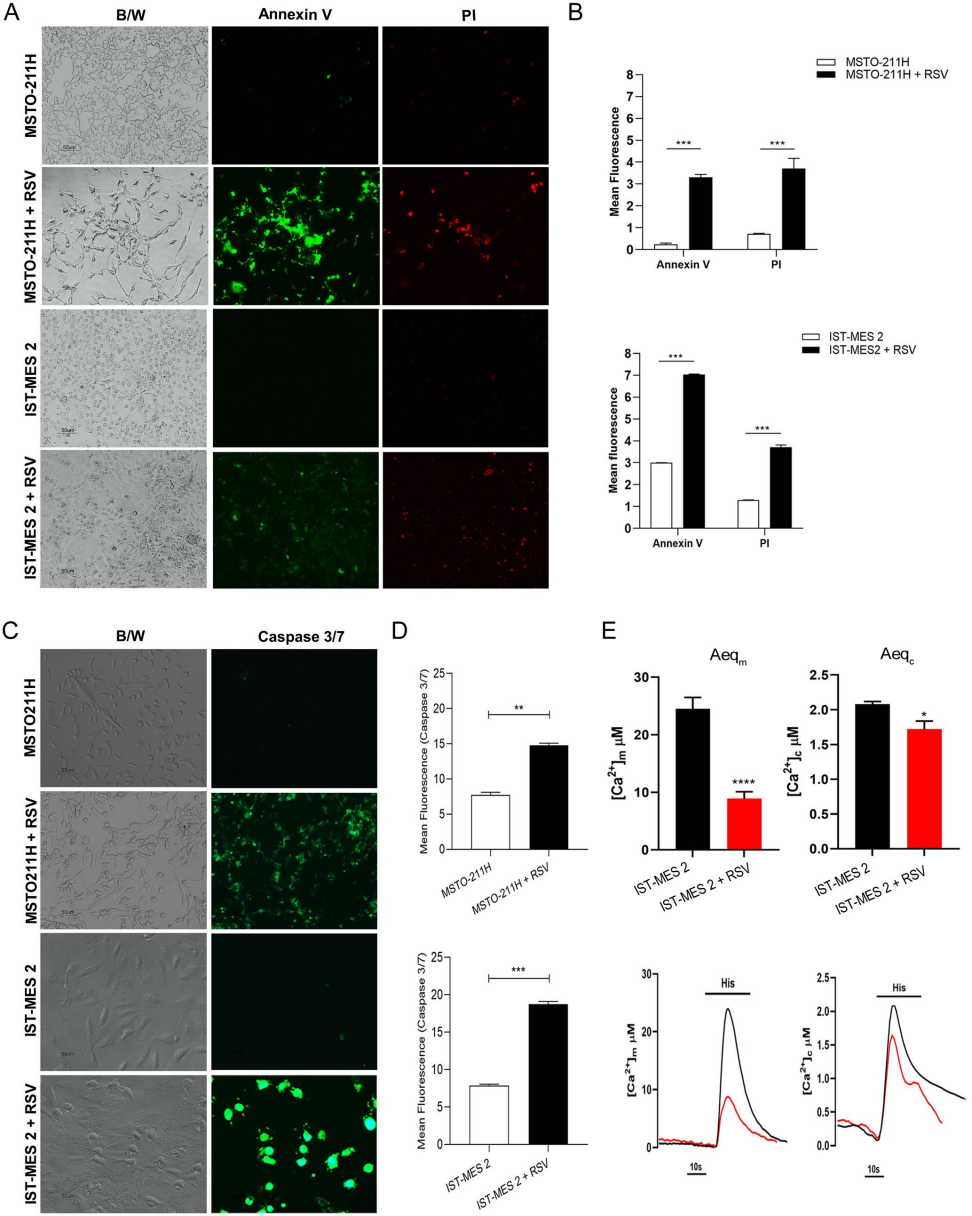
RSV induce apoptosis process on PM cells lines. (A) Annexin‐V (green)/PI (red) staining and (B) relative quantification in PM cell lines treated with RSV 100 μM for 48 h. (C) Immunostaining of activated caspase‐3/7 and (D) relative quantification in MSTO‐211H and IST‐MES 2 cells grown with RSV 100 μM for 48 h. (E) Variations in cytosolic and mitochondrial Ca²⁺ dynamics in IST‐MES‐2 cells treated with 100 μM RSV for 24 h, following histamine stimulation. Aeqm: mitochondrial calcium; Aeqc: cytosolic calcium. Representative traces are shown below. (Unpaired *t* test. *p < 0.05, ***p < 0.0001).

To test the potential of RSV to induce apoptosis in PM cells, two different methods were employed. Annexin V‐positive staining was observed in both PM cell lines exposed to RSV 100 μM (Figure 2A), suggesting that RSV induces activation of the apoptotic process. These data were also confirmed by Annexin V/PI fluorescence quantification, as reported in Figure 2B.

Activated Caspase‐3/7 expression was visualized in PM cells treated with RSV. Indeed, the Caspase 3/7‐specific fluorochrome revealed significantly higher levels of caspase 3/7‐positive cells (green) in PM cell lines exposed to RSV compared to untreated PM cells (Figure 2C), along with relative quantification (Figure 2D). Overall, these results demonstrate the pro‐apoptotic effects of RSV at 100 μM in both PM cell lines.

### 3.3 RSV Modulates Ca^2+^ Homeostasis in IST‐MES 2 Cells

Cellular Ca^2+^ signaling contributes to regulating apoptosis in tumor cells and tissues. In our study, intracellular Ca^2+^ signaling variations were analyzed in PM cell lines 24 h after treatment with RSV 100 μM. To measure Ca^2+^ concentration, aequorin probes targeting specific cellular localizations were used, such as the exclusively cytosolic wild‐type aequorin (cytAEQ) and the mitochondria‐targeted aequorin (mtAEQ). The addition of a hydrophobic prosthetic group (coelenterazine) to the plasmid allows the formation of an active probe. Coelenterazine can freely permeate the cell membrane and bind to aequorin. The binding of Ca^2+^ ions to three high‐affinity sites on aequorin causes oxidation of the prosthetic group and release of a photon. Aequorin luminescence data were calibrated offline into [Ca^2+^] values using a computer algorithm based on wild‐type and mutant aequorin Ca^2+^ response curves.

In our study, the MSTO‐211H cell line was more sensitive to RSV treatment; indeed, Ca^2+^ measurements could not be performed in MSTO‐211H cells. On the other hand, data show a significant reduction in mitochondrial [Ca^2+^] uptake 24 h after treatment with RSV 100 μM in response to histamine stimulation of IST‐MES 2 cells (****p < 0.0001). Similarly, a significant decrease in cytoplasmic [Ca^2+^] was recorded in these cells after RSV treatment (*p < 0.05) (Figure 2E). These data indicate that RSV treatment reduces both mitochondrial and cytosolic Ca²⁺ levels in IST‐MES‐2 cells, suggesting that RSV modulates intracellular Ca²⁺ signaling and may influence apoptosis through mechanisms independent of classical ER‐to‐mitochondria Ca²⁺ transfer.

### 3.4 RSV Inhibits Cell Migration in PM Cells

**FIGURE 3:**
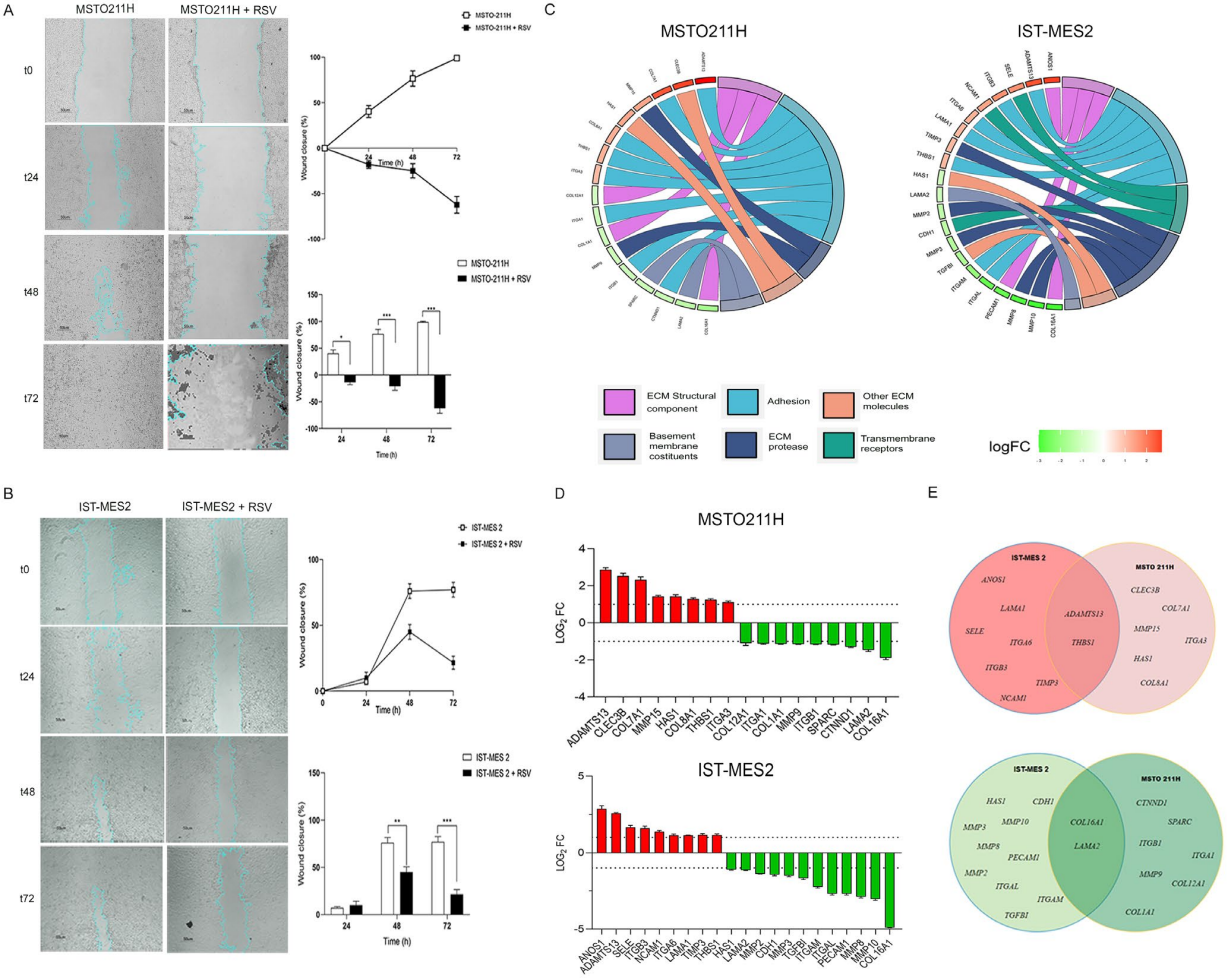
Extracellular matrix and adhesion molecules genes and cell migration modulated by RSV treatment in PM cells. (A, B) Wound‐healing assay on PM cell lines following RSV treatment at 24, 48 and 72 h. The wound closure rate was measured by detecting the closure distance at the time indicated in MSTO‐211H and IST‐MES 2 cell lines treated with RSV 100 μM. Representative micrographs under a phase contrast microscope are shown. Scale bar, 200 μm. Quantification of wound gap distance. Data are presented as the mean ± standard deviation (*n* = 3). (C) GO plots show the main pathways influenced by RSV treatment after 24 h. (D) *n* = 17 differentially expressed genes (DEGs), including *n* = 8 up‐regulated genes (> 1 log_2_ fold change; *p* < 0.05; red) and *n* = 9 down‐regulated genes (< −1 log_2_ fold change; *p* < 0.05; green), were identified in MSTO‐211H treated with RSV 100μM, compared to control. In IST‐MES 2, *n* = 21 differentially expressed genes, including *n* = 9 up‐regulated genes (> 1 log_2_ fold change; *p* < 0.05; red) and *n* = 12 down‐regulated genes (< −1 log_2_ fold change; *p* < 0.05; green), were identified compared to control. (E) Venn‐diagrams show the common DEGs in PM cell lines after RSV treatment (up‐regulated genes, upper graph; down‐regulated genes, lower graph).

Mesothelial cell line migration was analyzed to evaluate the anticancer potential of RSV 100 μM at 24, 48, and 72 h. To measure migratory capability, a wound healing assay was performed. Results showed that RSV significantly inhibited the ability of cells to close the gap in both PM cell lines (Figure 3A,B). In contrast, in untreated PM cells (control), the wound gap was closed by the end of the time course. The wound closure rate was measured by detecting the closure distance at each time point in MSTO‐211H and IST‐MES 2 cells treated with RSV 100 μM.

At 24 h, when most cells were still viable in both cell lines, a clear inhibitory effect of RSV on migration was evident. At 48 h, the inhibitory effect was more pronounced but should be interpreted considering that inhibition of proliferation, as assessed by MTT (≈50% reduction in viability in both cell lines), already contributed to the reduced migration. At 72 h, the widening of the wound was primarily attributable to the antiproliferative action of RSV rather than to migration inhibition. Therefore, while 24 h data mainly reflect a migration‐specific effect, the 48–72 h time points highlight the combined impact of reduced proliferation and impaired migration.

### 3.5 RSV Modulates ECM Expression Genes in PM Cell Lines

To better characterize the effects of RSV treatment on cell adhesion, the gene expression profiles of ECM and adhesion molecules were investigated using PCR array technology in PM cell lines (Figure 3, Tables 1 and 2). Down‐regulated genes (< −1 log2 FC; *p* < 0.05; green) and up‐regulated genes (> 1 log2 FC; *p* < 0.05; red) were identified in MSTO‐211H and IST‐MES2 cells exposed to RSV 100 μM for 48 h, compared to controls (Figure 3C, Tables 1 and 2).

**TABLE 1:** List of genes found to be *up‐regulated and down‐regulated* in MSTO‐211H cells treated with Resveratrol at 24 h.

Up‐regulated genes			Down‐regulated genes		
Number	Symbol/Acronym	Fold‐change (Log_2_ FC)	Number	Symbol/Acronym	Fold‐change (Log_2_ FC)
1	*ADAMTS13*	2,95	1	*COL16A1*	‐1,83
2	*CLEC3B*	2,64	2	*LAMA2*	‐1,42
3	*COL7A1*	2,44	3	*CTNND1*	‐1,26
4	*MMP15*	1,39	4	*SPARC*	‐1,16
5	*HAS1*	1,35	5	*ITGB1*	‐1,15
6	*COL8A1*	1,26	6	*MMP9*	‐1,12
7	*THBS1*	1,21	7	*COL1A1*	‐1,11
8	*ITGA3*	1,08	8	*ITGA1*	‐1,10
			9	*COL12A1*	‐1,00

Abbreviations: *ADAMTS13*, ADAM Metallopeptidase with Thrombospondin Type 1 Motif 13; *CLEC3B*, C‐Type Lectin Domain Family 3 Member B; COL12A1, Collagen Type XII Alpha 1 Chain; *COL16A1*, Collagen Type XVI Alpha 1 Chain; *COL1A1*, Collagen Type I Alpha 1 Chain; *COL7A1*, Collagen Type VII Alpha 1 chain; COL8A1, Collagen Type VIII Alpha 1 Chain; *CTNND1*, Catenin Delta 1; *HAS1*, Hyaluronan Synthase 1; *ITGA1*, Integrin Subunit Alpha 1; *ITGA3*, Integrin Subunit Alpha 3; *ITGB1*, Integrin Subunit Beta 1; *LAMA2*, Laminin Subunit Alpha 2; *MMP15*, Matrix metalloproteinase 15; *MMP9*, Matrix metalloproteinase 9; *SPARC*, Secreted Protein Acidic And Cysteine Rich; *THBS1*, Thrombospondin 1.

**TABLE 2:** List of genes found to be *up‐regulated and down‐regulated* in IST‐MES 2 treated with Resveratrol at 24 h.

Up‐regulated genes			Down‐regulated genes		
Number	Symbol/Acronym	Fold‐change (Log_2_ FC)	Number	Symbol/Acronym	Fold‐change (Log_2_ FC)
1	*ANOS1*	2,71	1	*COL16A1*	‐4,92
2	*ADAMTS13*	2,55	2	*MMP10*	‐3,09
3	*SELE*	1,75	3	*MMP8*	‐2,94
4	*ITGB3*	1,70	4	*PECAM1*	‐2,74
5	*NCAM1*	1,43	5	*ITGAL*	‐2,74
6	*ITGA6*	1,19	6	*ITGAM*	‐2,19
7	*LAMA1*	1,13	7	*TGFBI*	‐1,74
8	*TIMP3*	1,12	8	*MMP3*	‐1,56
9	*THBS1*	1,10	9	*CDH1*	‐1,49
			10	*MMP2*	‐1,39
			11	*LAMA2*	‐1,16
			12	*HAS1*	‐1,13

Abbreviations: *ADAMTS13*, ADAM Metallopeptidase with Thrombospondin Type 1 Motif 13; *ANOS1*, Anosmin 1; *CDH1*, Cadherin‐1; *COL16A1*, Collagen Type XVI Alpha 1 Chain; *HAS1*, Hyaluronan Synthase 1; *ITGA6*, Integrin Subunit Alpha 6; *ITGAL*, Integrin Subunit Alpha L; *ITGAM*, Integrin Subunit Alpha M; *ITGB3*, Integrin Subunit Beta 3; *LAMA1*, Laminin Subunit Alpha 1; *LAMA2*, Laminin Subunit Alpha 2; *MMP10*, Matrix Metallopeptidase 10; *MMP2*, Matrix Metallopeptidase 2; *MMP3*, Matrix Metallopeptidase 3; *MMP8*, Matrix Metallopeptidase 8; *NCAM1*, Neural Cell Adhesion Molecule 1; *PECAM1*, Platelet And Endothelial Cell Adhesion Molecule 1; *SELE*, Selectin E; *TGFBI*, Transforming Growth Factor Beta Induced; *THBS1*, Thrombospondin 1; *TIMP3*, TIMP Metallopeptidase Inhibitor 3.

Differentially Expressed Genes (DEGs; *n* = 17) in MSTO‐211H included 8 up‐regulated genes: ADAM Metallopeptidase with Thrombospondin Type 1 Motif 13 (ADAMTS13), C‐Type Lectin Domain Family 3 Member B (CLEC3B), Collagen Type I Alpha 1 Chain (COL1A1), Collagen Type VII Alpha 1 Chain (COL7A1), Matrix Metalloproteinase 15 (MMP15), Hyaluronan Synthase 1 (HAS1), Collagen Type VIII Alpha 1 Chain (COL8A1), Thrombospondin 1 (THBS1), Integrin Subunit Alpha 3 (ITGA3); and 9 down‐regulated genes: Collagen Type XVI Alpha 1 Chain (COL16A1), Laminin Subunit Alpha 2 (LAMA2), Catenin Delta 1 (CTNND1), Secreted Protein Acidic and Cysteine Rich (SPARC), Integrin Subunit Beta 1 (ITGB1), Matrix Metalloproteinase 9 (MMP9), Collagen Type I Alpha 1 Chain (COL1A1), Integrin Subunit Alpha 1 (ITGA1), and Collagen Type XII Alpha 1 Chain (COL12A1) (Table 1).

In IST‐MES‐2 cells treated with RSV 100 μM, DEGs (*n* = 21) included 9 up‐regulated genes: Anosmin 1 (ANOS1), Thrombospondin Type 1 Motif 13 (ADAMTS13), Selectin E (SELE), Integrin Subunit Beta 3 (ITGB3), Neural Cell Adhesion Molecule 1 (NCAM1), Integrin Subunit Alpha 6 (ITGA6), Laminin Subunit Alpha 1 (LAMA1), Metallopeptidase Inhibitor 3 (TIMP3), Thrombospondin 1 (THBS1); and 12 down‐regulated genes: Collagen Type XVI Alpha 1 Chain (COL16A1), Matrix Metallopeptidase 10 (MMP10), Platelet and Endothelial Cell Adhesion Molecule 1 (PECAM1), Integrin Subunit Alpha L (ITGAL), Integrin Subunit Alpha M (ITGAM), Transforming Growth Factor Beta Induced (TGFB1), Matrix Metallopeptidase 3 (MMP3), Cadherin‐1 (CDH1), Matrix Metallopeptidase 2 (MMP2), Laminin Subunit Alpha 2 (LAMA2), Hyaluronan Synthase 1 (HAS1) (Table 2).

### 3.6 RSV Modifies the PM Cell Morphology and Affects the Expression of E‐Cadherin and TGF‐β Proteins, as Well as the Localization of β Catenin in PM Cell Lines

**FIGURE 4:**
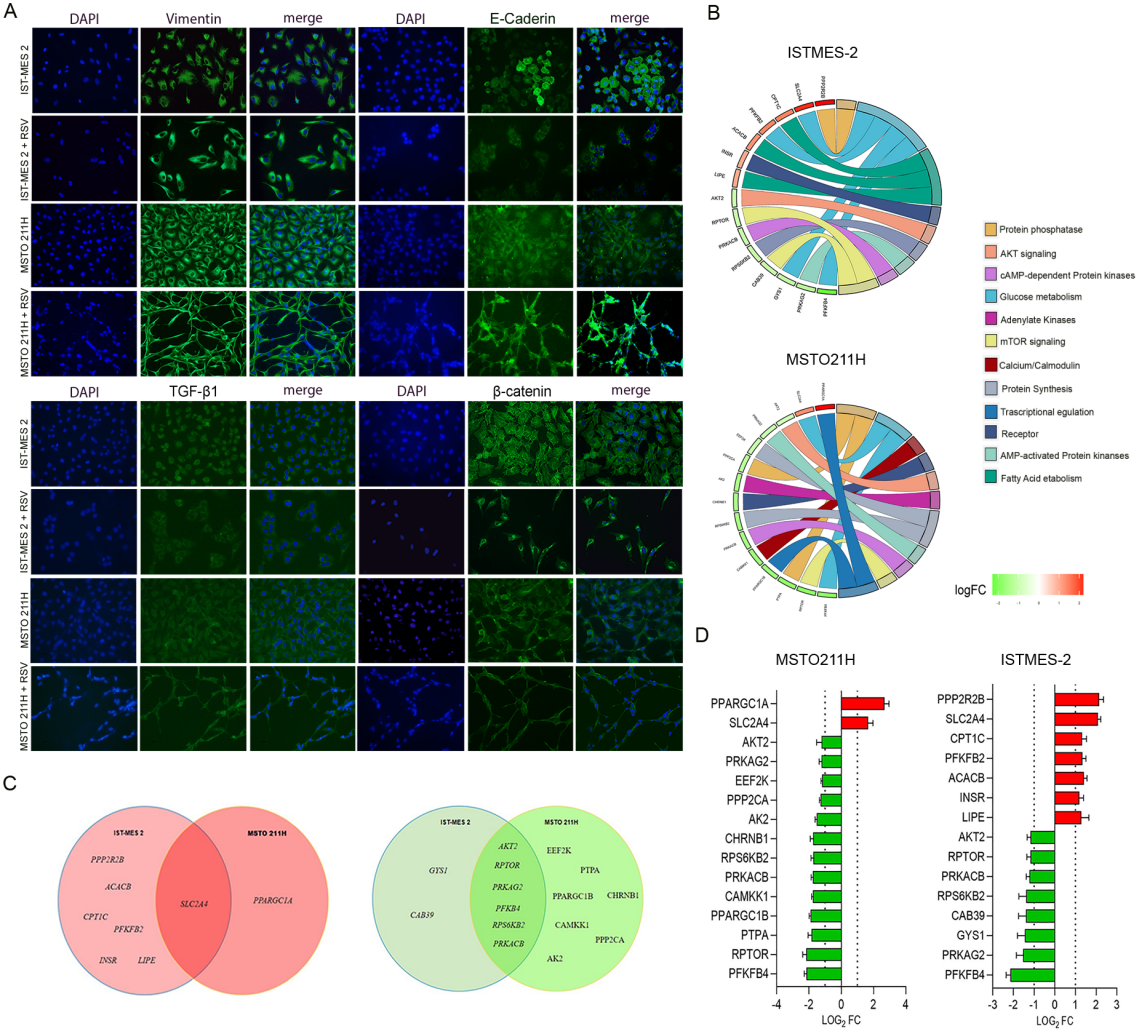
RSV treatment affects the expression of proteins involved in EMT process and genes related to cellular metabolism. (A) The panel show the effect of RSV treatment on Vimentin, E‐cadherin, TGF‐b1 and b‐catenin in PM cell lines. (B) GO plots show the main pathways influenced by RSV treatment after 24 h. (C) Venn‐diagrams show the common DEGs in PM cell lines after RSV treatment (up‐regulated genes, left graph; down‐regulated genes, right graph). (D) *n* = 15 differentially expressed genes, including *n* = 2 up‐regulated genes (> 1 log_2_ fold change; *p* < 0.05; red) and *n* = 13 down‐regulated genes (< −1 log_2_ fold change; *p* < 0.05; green), were identified in MSTO‐211H treated with RSV 100μM, compared to control. In IST‐MES 2, *n* = 15 differentially expressed genes, including *n* = 7 up‐regulated genes (> 1 log_2_ fold change; *p* < 0.05; red) and *n* = 8 down‐regulated genes (< −1 log_2_ fold change; *p* < 0.05; green), were identified compared to control.

MSTO‐211H and IST‐MES 2 represent biphasic and epithelioid PM cell subtypes, respectively. Specifically, the epithelioid histotype is usually composed of round epithelioid cells, typically displaying eosinophilic cytoplasm and round nuclei with small nucleoli. Biphasic PM has a characteristic pattern containing a mixture of epithelioid and sarcomatoid areas within the same tumor; cells in the latter appear elongated with a spindle shape. Immunofluorescence assay for vimentin protein revealed that, in both PM cell lines, RSV treatment modified cell morphology and cytoskeleton organization compared to controls (Figure 4A).

Our results showed that RSV 100 μM influenced E‐cadherin protein expression in the IST‐MES 2 cell line. Specifically, a loss of cell–cell interaction was observed in IST‐MES 2 cells treated with RSV compared to control (Figure 4A). At the protein level, TGF‐β expression appeared unaltered by RSV treatment in IST‐MES 2 cells compared to control (Figure 4A).

RSV prevented nuclear accumulation of β‐catenin. As shown in Figure 4A, MSTO‐211H and IST‐MES2 cells were evaluated for β‐catenin cellular localization. Immunofluorescence suggested that RSV blocked the translocation of β‐catenin into the nucleus in IST‐MES 2 cells, thereby inhibiting the WNT pathway. Conversely, in untreated PM cell lines, β‐catenin was found in both the cytoplasm and nuclei (Figure 4A). Generally, digital images showed altered cellular morphology in MSTO‐211H cells treated with RSV, along with nuclear disruption (Figure 4A).

### 3.7 RSV Modulates the AMPK Signaling in PM Cells

The AMPK gene expression profile was investigated using PCR array technology in PM cell lines treated with RSV (Figure 4B–D, Tables 3 and 4). Gene Ontology (GO) analysis showed that RSV modulated genes involved in several signaling pathways, as reported in Figure 4B. Specifically, differentially expressed genes (DEGs; *n* = 15) were identified in IST‐MES 2 cells, including 7 up‐regulated genes (> 1 log2 FC; *p* < 0.05; red) and 8 down‐regulated genes (< −1 log2 FC; *p* < 0.05; green) (Figure 4C,D and Table 3).

**TABLE 3:** List of genes found to be *upregulated and downregulated* in IST‐MES 2 treated with Resveratrol at 24 h.

Up‐regulated genes			Down‐regulated genes		
Number	Symbol/Acronym	Fold‐Change (Log_2_ FC)	Number	Symbol/Acronym	Fold‐Change (Log_2_ FC)
1	*PPP2R2B*	2,30	1	*PFKB4*	−2,29
2	*SLC2A4*	2,18	2	*PRKAG2*	−1,30
3	*CPT1C*	1,47	3	*GYS1*	−1,19
4	*PFKFB2*	1,46	4	*CAB39*	−1,14
5	*ACACB*	1,30	5	*RPS6KB2*	−1,13
6	*INSR*	1,04	6	*PRKACB*	−1,11
7	*LIPE*	1,03	7	*RPTOR*	−1,05
			8	*AKT2*	−1,05

Abbreviations: *ACACB*, Acetyl‐CoA Carboxylase Beta; *AKT2*, AKT Serine/Threonine Kinase 2; *CAB39*, Calcium Binding Protein 39; *CPT1C*, Carnitine Palmitoyltransferase 1C; *GYS1*, Glycogen Synthase 1; *INSR*, Insulin Receptor; *LIPE*, Lipase E, Hormone Sensitive Type; *PFKB4*, 6‐Phosphofructo‐2‐Kinase/Fructose‐2,6‐Bisphosphatase 4; *PFKFB2*, Phosphofructo‐2‐Kinase/Fructose‐2,6‐Bisphosphatase 2; *PPP2R2B*, Protein phosphatase 2 regulatory subunit Bbeta; *PRKACB*, Protein Kinase CAMP‐Dependent Catalytic Subunit Beta; *PRKAG2*, Protein Kinase AMP‐Activated Non‐Catalytic Subunit Gamma 2; *RPS6KB2*, Ribosomal Protein S6 Kinase B2; *RPTOR*, Regulatory‐Associated Protein of MTOR; *SLC2A4*, Solute Carrier Family 2 Member 4.

**TABLE 4:** List of genes found to be *up‐regulated and down‐regulated* in MSTO‐211H cells treated with Resveratrol at 24 h.

Up‐regulated genes			Down‐regulated genes		
Number	Symbol/Acronym	Fold‐change (Log_2_ FC)	Number	Symbol/Acronym	Fold‐change (Log_2_ FC)
1	*PPARGC1A*	2,49	1	*PFKB4*	−2,06
2	*SLC2A4*	1,44	2	*RPTOR*	−2,03
			3	*PTPA*	−1,99
			4	*PPARGC1B*	−1,85
			5	*CAMKK1*	−1,81
			6	*PRKACB*	−1,69
			7	*RPS6KB2*	−1,63
			8	*CHRNB1*	−1,62
			9	*AK2*	−1,59
			10	*PPP2CA*	−1,33
			11	*EEF2K*	−1,13
			12	*PRKAG2*	−1,12
			13	*AKT2*	−1,01

Abbreviations: *AK2*, Adenylate Kinase 2; *AKT2*, Protein Kinase B Beta (AKT Serine/Threonine Kinase 2); *CAMKK1*, Calcium/Calmodulin‐Dependent Protein Kinase Kinase 1; *CHRNB1*, Cholinergic Receptor Nicotinic Beta 1 Subunit; *EEF2K*, Eukaryotic Elongation Factor 2 Kinase; *PFKB4*, 6‐Phosphofructo‐2‐Kinase/Fructose‐2,6‐Biphosphatase 4; *PPARGC1A*, Peroxisome Proliferator‐Activated Receptor Gamma Coactivator 1 Alpha; *PPARGC1B*, Peroxisome Proliferator‐Activated Receptor Gamma Coactivator 1 Beta; *PPP2CA*, Protein Phosphatase 2 Catalytic Subunit Alpha; *PRKACB*, Protein Kinase, cAMP‐Dependent, Catalytic Subunit Beta; *PRKAG2*, Protein Kinase AMP‐Activated Non‐Catalytic Subunit Gamma 2; *PTPA*, Protein Phosphatase 2 Phosphatase Activator; *RPS6KB2*, Ribosomal Protein S6 Kinase B2; *RPTOR*, Regulatory‐Associated Protein of MTOR Complex 1; *SLC2A4*, Solute Carrier Family 2 Member 4 or GLUT4, Glucose Transporter.

In IST‐MES 2 cells, the DEGs implicated in glucose metabolism included up‐regulated genes such as Solute Carrier Family 2 Member 4 (SLC2A4), Protein Phosphatase 2 Regulatory Subunit B (PFKFB2), and Insulin Receptor (INSR), while Glycogen Synthase 1 (GYS1) and 6‐Phosphofructo‐2‐Kinase/Fructose‐2,6‐Bisphosphatase 4 (PFKB4) were down‐regulated. Other up‐regulated genes involved in fatty acid metabolism included Acetyl‐CoA Carboxylase Beta (ACACB), Carnitine Palmitoyltransferase 1C (CPT1C), Lipase E, Hormone Sensitive Type (LIPE), and Protein Phosphatase 2 Regulatory Subunits Beta (PPP2R2B).

RSV treatment in IST‐MES 2 also produced down‐regulation of genes implicated in AKT and PI3 Kinase signaling, including AKT Serine/Threonine Kinase 2 (AKT2), AMP‐Activated Protein Kinase subunit Gamma 2 (PRKAG2), and cAMP‐Dependent Protein Kinase Catalytic Subunit Beta (PRKACB). Furthermore, RES induced down‐regulation of genes involved in mTOR signaling, such as Calcium Binding Protein 39 (CAB39), Regulatory‐Associated Protein of MTOR (RPTOR), and Ribosomal Protein S6 Kinase B2 (RPS6KB2).

In MSTO‐211H cells, 15 DEGs were identified following RSV treatment (100 μM), including 2 up‐regulated genes (> 1 log2 FC; *p* < 0.05; red) and 13 down‐regulated genes (< −1 log2 FC; *p* < 0.05; green) (Figure 4C,D and Table 4). RSV treatment resulted in down‐regulation of genes involved in AKT and PI3 Kinase signaling, Calcium/Calmodulin signaling, cAMP‐Dependent Protein Kinases, and mTOR signaling. Specifically, adenylate kinase 2 (AK2), Protein Kinase B Beta (AKT2), and Calcium/Calmodulin‐Dependent Protein Kinase Kinase 1 (CAMKK1) were down‐regulated, affecting their respective signaling pathways.

Additionally, cAMP‐dependent catalytic subunit beta (PRKACB) and Regulatory‐Associated Protein of MTOR Complex 1 (RPTOR) were down‐regulated. The AMP‐Activated Protein Kinase subunit Gamma 2 (PRKAG2) was also down‐regulated.

Genes involved in glucose metabolism showed mixed regulation: Glucose Transporter (SLC2A4) was up‐regulated, while Phosphofructo‐2‐Kinase/Fructose‐2,6‐Bisphosphatase 4 (PFKB4) was down‐regulated compared to control. Protein phosphatase molecules, such as Protein Phosphatase 2 Catalytic Subunit Alpha (PPP2CA), were down‐regulated. Proteins involved in protein synthesis, including Eukaryotic Elongation Factor 2 Kinase (EEF2K) and Ribosomal Protein S6 Kinase B2 (RPS6KB2), were also down‐regulated.

Transcriptional regulation proteins showed differential expression: Proliferator‐Activated Receptor Gamma Coactivator 1 Alpha (PPARGC1A) was up‐regulated, while Proliferator‐Activated Receptor Gamma Coactivator 1 Beta (PPARGC1B) was down‐regulated. Non‐catalytic protein phosphatase subunits, such as Protein Phosphatase 2 Phosphatase Activator (PTPA) and Cholinergic Receptor Nicotinic Beta 1 Subunit (CHRNB1), were both down‐regulated in MSTO‐211H cells treated with RSV.

## 4 Discussion

The results of the present study indicate that RSV is able to affect PM cancer properties by modulating cell proliferation in a time‐ and dose‐dependent manner. Initially, our results demonstrated that RSV reduces proliferation of PM cell lines and, simultaneously, inhibits their migratory ability, as shown by the wound‐healing assay. Moreover, our data revealed that RSV treatment induces apoptosis through activation of Caspase‐3/7.

Cellular Ca^2+^ signaling is also involved in regulating many processes, including apoptosis. Specifically, the endoplasmic reticulum transmits precise Ca^2+^ signals to mitochondria, which interpret them as specific inputs to regulate essential functions such as metabolism, energy production, and apoptosis [19]. Our results highlight that treatment with 100 μM RSV alters intracellular Ca²⁺ signaling and may trigger the activation of several fundamental cellular processes, including apoptosis. Despite the observed reduction in mitochondrial Ca²⁺ levels, apoptotic markers are activated, suggesting that RSV can induce apoptosis through mechanisms at least partially independent of mitochondrial Ca²⁺ signaling. The concentration of Ca²⁺ at the mitochondrial level could indicate that mitochondria remain critical modulators of apoptosis, even when their activation is influenced by alternative, Ca²⁺‐independent pathways. Although the 100 μM concentration of RSV exceeds the plasma levels achievable in humans, it was selected based on our dose–response data, where it showed significant antiproliferative and pro‐apoptotic effects in both PM cell lines. This concentration is consistent with other in vitro cancer studies, where similar doses are commonly used to compensate for RSV's poor bioavailability and to allow a clearer investigation of its molecular mechanisms of action. Indeed, the oral bioavailability of RSV is limited due to rapid metabolism and clearance, which explains why clinical trials often administer very high doses (up to several grams per day) to reach therapeutically relevant tissue concentrations [23, 24]. Moreover, in vitro studies typically require higher concentrations to model the local effects in tumor tissues and to overcome the absence of active metabolites, justifying our use of 100 μM to better assess the compound's efficacy in mesothelioma cells. However, precisely because of the poor oral bioavailability of RSV, promising strategies are being developed to improve its absorption and therapeutic efficacy in humans, including the use of liposomes, cyclodextrins, solid lipid nanoparticles, and polymeric micelles [25, 26, 27]. Furthermore, more well‐controlled clinical trials with larger patient cohorts and a better understanding of the pharmacokinetics and pharmacodynamics of the compound are needed to prove the efficacy of RSV in cancer patients and to identify the optimal formulation required to achieve therapeutic concentrations in humans.

In addition to the effects observed with RSV alone, we further explored its potential as an adjuvant by testing its combination with an A3 adenosine receptor (A3AR) agonist. Previous studies have highlighted A3AR as a promising therapeutic target in mesothelioma, given its ability to trigger anti‐proliferative and pro‐apoptotic pathways [28, 29]. Consistently, our results showed that RSV synergistically potentiates the pro‐apoptotic activity of Cl‐IB‐MECA at low concentrations (1 nM) in MSTO‐211H cells. The co‐treatment markedly increased Caspase 3/7 activation and Annexin V positivity compared to Cl‐IB‐MECA alone (Figure S1), further supporting the rationale for combining RS with A3AR‐targeting agents to enhance therapeutic efficacy in PM.

Gene expression analyses revealed that RSV interferes with the expression of genes involved in cell–cell and cell‐ECM adhesion. In our model, COL1A1 expression was downregulated in PM cells treated with RSV. Notably, high COL1A1 expression in PM tissues has been associated with diagnosis, prognosis, and disease progression [30]. Similarly, survival analysis using TCGA data showed that high COL1A1 and COL4A2 expression levels correlate with poor survival in PM patients [6]. RSV treatment also led to downregulation of COL16A1 in both MSTO‐211H and IST‐MES2, and of CTNND1 in MSTO‐211H. In endometrial carcinoma, miR‐543 and miR‐1271‐5p downregulate COL16A1 [31] and CTNND1 [32], respectively, inhibiting proliferation, migration, and invasion.

Interestingly, RSV induced upregulation of ADAMTS13 in PM cell lines. Low ADAMTS13 expression has been associated with poor survival in various cancers, including lung cancer [33, 34]. In IST‐MES 2, it has been observed a decrease of TGF‐β after RSV treatment. TGF‐β signaling has been extensively associated with lung cancer development [34]. RSV treatment inhibited LAMA2 expression in PM cells. Consistently, LAMA2 knockdown was shown to restore cell migration and PI3K‐AKT pathway activation in lung adenocarcinoma [35]. SPARC, a gene involved in ECM synthesis, tumor invasion, and EMT, is also implicated in promoting cancer aggressiveness [36]. Matrix metalloproteinases (MMPs), key players in tumor progression [37], were downregulated by RSV: MMP‐2, −3, and −10 in IST‐MES 2, and MMP‐9 in MSTO‐211H.

RSV also altered cell morphology, as indicated by vimentin staining, and affected EMT‐related proteins such as β‐catenin and E‐cadherin in IST‐MES2. Notably, CDH1 was downregulated in IST‐MES2 after RSV treatment, supporting its relative resistance to RSV compared to MSTO‐211H. RSV also influenced cellular metabolism, acting on the AMPK pathway. Among the differentially expressed genes, PPP2CA was downregulated in MSTO‐211H. PPP2CA, encoding the catalytic subunit of PP2A, is overexpressed in several cancers and linked to poor prognosis [38, 39]. PFKFB4, which regulates glycolysis and ATP synthesis, was also downregulated by RES. PFKFB4 is highly expressed in many cancers, and its inhibition may impair tumor energy metabolism [40].

RPS6KB2, a downstream effector of mTOR overexpressed in various cancers [41], was downregulated in PM cells following RSV treatment. RSV also induced upregulation of CPT1C in IST‐MES2. CPT1C, upregulated under metabolic stress, modulates fatty acid homeostasis and energy balance in tumor cells [42]. Additionally, PPARGC1A, LIPE, and ACACB—genes involved in lipid metabolism—were upregulated in IST‐MES‐2, while PPARGC1B was downregulated in MSTO‐211H. PPARGC1A and PPARGC1B regulate key metabolic processes, including adipogenesis, lipolysis, mitochondrial biogenesis, and gluconeogenesis [39, 43, 44, 45].

RSV significantly downregulated AKT2 expression in MSTO‐211H. AKT2, a key component of the PI3K/AKT pathway, is frequently overexpressed in various cancers and promotes proliferation and metastasis [46, 47, 48]. RSV may inhibit AKT2 by interacting with its ATP‐binding site, suppressing downstream targets like mTOR and FOXO transcription factors [49, 50, 51]. This aligns with previous studies showing that AKT2 knockdown reduces proliferation and induces apoptosis [49, 52].

Interestingly, previous studies have shown that RSV activates the AMPK pathway while inhibiting MAPK and PI3K/AKT signaling, all of which are critically involved in cancer development and progression [52]. Adenylate kinase 2 (AK2), a mitochondrial isoenzyme of the AK family, plays a key role in cellular energy metabolism and signal transduction [53, 54]. In our study, RSV treatment at 100 μM led to downregulation of AK2 expression in MSTO‐211H cells. This is noteworthy given AK2's recognized oncogenic role in several cancers, including lung adenocarcinoma [51]. AK2's localization in the mitochondrial intermembrane space highlights its role in maintaining the ATP/ADP balance between mitochondria and cytoplasm—an essential aspect of cellular energy homeostasis [55]. Disruption of this balance has been implicated in the pathogenesis of various diseases, including cancer [53]. In lung adenocarcinoma, AK2 overexpression correlates with tumor progression and poor patient survival [51]. Functional studies have demonstrated that AK2 knockdown in lung adenocarcinoma cell lines suppresses proliferation, migration, and invasion, while promoting apoptosis and autophagy, underscoring its role in tumor aggressiveness and metastatic potential [51]. These findings align with evidence linking AK2 dysregulation to multiple cancers, including PM [56, 57, 58]. Moreover, AK2 has been implicated in modulating epithelial‐mesenchymal transition (EMT), a key process in metastasis, as shown in lung adenocarcinoma models [51]. Therefore, the observed downregulation of AK2 in PM cells following RSV treatment may represent a mechanism through which RSV inhibits EMT, thereby reducing tumor cell migration and invasiveness.

In our study, qPCR array analysis revealed a significant upregulation of the insulin receptor (INSR) in IST‐MES 2 cells following RSV treatment, compared to untreated controls. This finding is particularly relevant given INSR's role in promoting tumor progression. One of the hallmarks of cancer is the ability of tumor cells to migrate and invade the extracellular matrix—key steps in metastasis [59, 60]. Within this context, INSR and its downstream effector, insulin receptor substrate‐1 (IRS‐1), are critical mediators of the insulin signaling pathway and are known to support tumorigenesis and cancer development [61, 62]. Together with the insulin‐like growth factor 1 receptor (IGF1R), INSR operates within the broader IGF signaling network to exert pro‐tumourigenic effects [63, 64]. INSR also plays a central role in regulating glucose metabolism, another hallmark of cancer. Notably, the PI3K/AKT signaling pathway, a major downstream branch of insulin signaling, governs key processes such as glycolysis, cellular metabolism, and proliferation [65]. Therefore, INSR may be instrumental in sustaining metabolic functions and the survival of tumor cells [66, 67, 68]. The observed INSR upregulation upon RSV treatment may represent a compensatory response aimed at counterbalancing RSV's pro‐apoptotic effects, thereby supporting cell survival in IST‐MES 2.

Further analysis revealed a significant downregulation of CAMKK1 in RSV‐treated MSTO‐211H cells. CAMKK1, a serine/threonine kinase, is a key regulator of calcium signaling and activates critical downstream effectors such as AMPK and Akt [69, 70]. The downregulation of CAMKK1 suggests a possible disruption in these pathways, which could underlie the anti‐proliferative and pro‐apoptotic effects of RSV. CAMKK1 has been linked to oncogenesis in several cancers. In lung cancer, a CAMKK1 gene variant is associated with increased cancer risk, and its knockdown has been shown to promote apoptosis through reduced Akt phosphorylation at Thr308, as observed in breast cancer models [69, 71]. Since phosphorylation at Thr308 is essential for Akt activation and for the inhibition of apoptotic proteins like caspase‐3/7 and caspase‐9, RSV‐induced downregulation of CAMKK1 may impair Akt signaling and promote apoptosis [69, 72]. Additionally, PRKACB expression was downregulated in both MSTO‐211H and IST‐MES 2 cell lines following RSV treatment. PRKACB encodes a catalytic subunit of protein kinase A (PKA), which plays a critical role in cAMP signaling. PRKACB has been identified as an oncogene involved in cancer progression, particularly in endocrine malignancies [73]. Thus, the suppression of PRKACB by RSV may contribute to its anti‐tumor effects in PM by interfering with PKA‐mediated signaling pathways.

## 5 Conclusion

In summary, RSV has been reported to exhibit both pro‐apoptotic and pro‐survival effects in cancer cells, depending on the specific cellular context and the dose used. Our results suggest that RSV could be considered a novel adjuvant treatment for PM, particularly for the biphasic histotype. This represents a significant step forward in developing alternative and more effective therapies for PM, especially given that standard treatments, such as chemotherapy, surgery, and radiotherapy, have yielded unsatisfactory outcomes.

As a future perspective, we plan to investigate the molecular mechanisms underlying RSV's antitumor effects in mesothelioma, focusing particularly on the functional roles of metabolic genes such as SLC2A4 and PFKFB4 through loss‐of‐function and rescue approaches. These studies will be complemented by in vivo experiments using relevant animal models to validate the translational potential of RSV, and by analyses of clinical samples to assess its therapeutic applicability and optimize treatment strategies.

## Author Contributions

**Maria Rosa Iaquinta:** writing – original draft. **Raffaella De Pace, Assia Benkhalqui, Cecilia Pesaresi, Simone Patergnani, Giulio Righes:** methodology. **Paolo Pinton:** writing – original draft. **Elisa Mazzoni:** conceptualization and writing – original draft, Supervision. **Fernanda Martini:** writing – original draft. **Mauro Tognon:** writing – original draft.

## Conflicts of Interest

The authors declare no conflicts of interest.

## Supporting information


**Figure S1:** Evaluation of apoptosis and necrosis processes in MSTO‐211H cells 48 h after treatment with 100 μM resveratrol (RSV), 1 nM Cl‐IB‐MECA, and the combination of 1 nM Cl‐IB‐MECA with 100 μM RSV. (A) Green fluorescence emission (Annexin V) indicating apoptosis and red fluorescence emission (Propidium Iodide) indicating late apoptosis and necrosis. Magnification 10X. (B) Quantification of green (Annexin) and red (PI) fluorescence. *p < 0.05; **p < 0.001; ***p < 0.0001 (two‐way ANOVA and Tukey test). (C) Positive expression of pro‐apoptotic proteins Caspases 3/7. Magnification 10X. (D) Quantification of fluorescence emitted by the cells. A significant increase in Caspase 3/7 was observed in the group treated with Cl‐IB‐MECA in combination with RSV. *p < 0.05; **p < 0.001; ***p < 0.0001 (two‐way ANOVA and Tukey test).

## Data Availability

The data that has been used is confidential.
