# Supplementary figures and images for: Resveratrol Affects Cell Activities, Induces Apoptosis and Regulates AMPK Signaling Pathway in Pleural Mesothelioma Cells
Source: FASEB J. 2025 Oct 19;39(20):e71120. doi: 10.1096/fj.202500657RR (PMC12535758; doi:10.1096/fj.202500657RR)

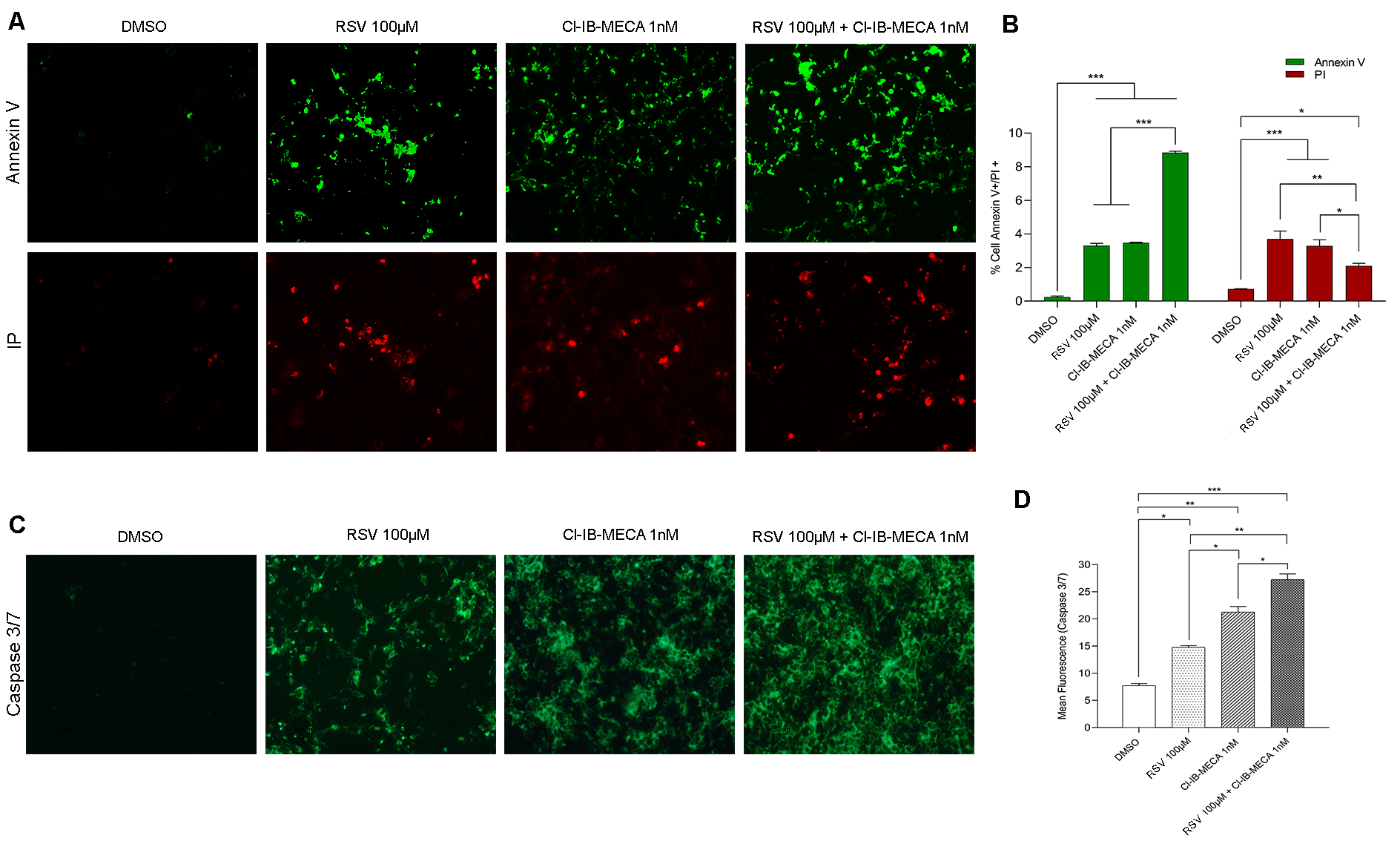

Supplement: Supplementary file 1 — Figure S1: Evaluation of apoptosis and necrosis processes in MSTO‐211H cells 48 h after treatment with 100 μM resveratrol (RSV), 1 nM Cl‐IB‐MECA, and the combination of 1 nM Cl‐IB‐MECA with 100 μM RSV. (A) Green fluorescence emission (Annexin V) indicating apoptosis and red fluorescence emission (Propidium Iodide) indicating late apoptosis and necrosis. Magnification 10X. (B) Quantification of green (Annexin) and red (PI) fluorescence. *p < 0.05; **p < 0.001; ***p < 0.0001 (two‐way ANOVA and Tukey test). (C) Positive expression of pro‐apoptotic proteins Caspases 3/7. Magnification 10X. (D) Quantification of fluorescence emitted by the cells. A significant increase in Caspase 3/7 was observed in the group treated with Cl‐IB‐MECA in combination with RSV. *p < 0.05; **p < 0.001; ***p < 0.0001 (two‐way ANOVA and Tukey test). [file FSB2-39-e71120-s001.tif]
